# Genetically Encoded Fluorescence Resonance Energy Transfer Biosensor for Live-Cell Visualization of Lamin A Phosphorylation at Serine 22

**DOI:** 10.34133/bmr.0091

**Published:** 2024-10-22

**Authors:** Jian Liu, Qianqian Li, Jinfeng Wang, Juhui Qiu, Jing Zhou, Qin Peng

**Affiliations:** ^1^ Shenzhen Bay Laboratory, Shenzhen 518132, China.; ^2^ Key Laboratory for Biorheological Science and Technology of Ministry of Education, State and Local Joint Engineering Laboratory for Vascular Implants, College of Bioengineering, Chongqing University, Chongqing 400030, China.; ^3^ Department of Physiology and Pathophysiology, School of Basic Medical Sciences, State Key Laboratory of Vascular Homeostasis and Remodeling, Department of Cardiology, Peking University Third Hospital, National Health Commission Key Laboratory of Cardiovascular Molecular Biology and Regulatory Peptides, Beijing Key Laboratory of Cardiovascular Receptors Research, Peking University, Beijing 100191, China.

## Abstract

Extensive phosphorylation at serine 22 (pSer22) on lamin A is the hallmark of cell mitosis, which contributes to the breakdown of nuclear envelope. In the interphase, pSer22 lamin A exists in low abundance and is involved in mechanotransduction, virus infection, and gene expression. Numerous evidences emerge to support lamin A regulation on cell function and fate by phosphorylation. However, live-cell imaging tools for visualizing the dynamics of pSer22 lamin A are yet to be established. Herein, we developed a novel lamin A phosphorylation sensor (LAPS) based on fluorescence resonance energy transfer (FRET) with high sensitivity and specificity. We observed the dynamic lamin A phosphorylation during the cell cycle progression in single living cells: the increase of pSer22 modification when cells entered the mitosis and recovered upon the mitosis exit. Our biosensor also showed the gradual reduction of pSer22 modification during cell adhesion and in response to hypotonic environment. By applying LAPS, we captured the propagation of pSer22 modification from inside to outside of the inner nuclear membrane, which further led to the breakdown of nuclear envelope. Meanwhile, we found the synchronous phosphorylation of pSer22 lamin A and H3S10ph at mitosis entry. Inhibition of Aurora B, the responsible kinase for H3S10ph, could shorten the mitotic period without obvious effect on the pSer22 modification level of lamin A. Thus, LAPS allows the spatiotemporal visualization of the lamin A pSer22, which will be useful for elucidating the molecular mechanisms underlying cell mitosis and mechanoresponsive processes.

## Introduction

Lamin A is the main component of lamina that supports the integration of nuclear envelope (NE) and highly associated with heterochromatin tethering at the nuclear periphery [1–3]. The protein level of lamin A on nuclear membrane determines different cell fate during differentiation [4–7]. The disassembly and degradation of lamin A is dependent on the phosphorylation events [8], catalyzed by numerous kinases, like CDK1, PKC, and CDK5 [9–11]. During mitosis, serine 22 of lamin A is extensively phosphorylated by CDK1 and PKC, inducing rapid breakdown of NE [12,13]. In the interphase, lamin A phosphorylation at serine 22 (pSer22) is maintained at a low level [14], but dynamically regulated in response to matrix stiffness [15,16] and virus infection [17]. Recently, pSer22 lamin A has been identified to interact with active enhancer and promote gene expression, which contributes to the up-regulation of progeria-related risky genes [18]. Moreover, lamin A gets phosphorylated at the DNA double-strand break site by ATM- and Rad3-related (ATR) kinase, which eventually induces NE rupture [19]. Although pSer22 modification on lamin A shows versatile functionality, it lacks live-cell visualization methods to spatiotemporally track the dynamics of pSer22 lamin A for better mechanistic understanding in regulating cell cycle and genomics.

Recently, a lamin A nanobody-based FRET biosensor was designed to indicate mechanical forces on nuclear lamin A [20]. The FRET biosensor contains 2 lamin A nanobodies at both N and C terminus flanking an existing FRET module, which could measure the intermolecular force between adjacent lamin A. The biosensor successfully visualized the variation of mechanical strain of lamin filaments after regulation of nuclear volume, functional LINC (linker of nucleoskeleton and cytoskeleton) complex, and actomyosin contractility. However, it cannot visualize whether lamin A is depolymerized or mediated by pSer22 in response to mechanical force.

Herein, we designed a novel lamin A phosphorylation sensor (LAPS) based on FRET to real-time visualize pSer22 modification on lamin A. By screening the lamin A targeting strategy and linker length, we obtained a biosensor that can indicate the pSer22 level on lamin A with high specificity and sensitivity. Using LAPS, we successfully observed the boost of pSer22 modification before the NE breakdown at the entrance of mitosis. Meanwhile, we observed gradual reduction of pSer22 modification during the cell adhesion and in response to hypotonic stimuli, which may correspond to the enhanced lamina stress. With high spatiotemporal resolution of LAPS, we found that pSer22 modification on lamin A that was located on NE occurred gradually from the inside to outside of the inner nuclear membrane when cells entered the mitosis. Concurrently with pSer22 modification on lamin A was the phosphorylation of serine 10 in histone 3 (H3S10ph) and chromatin condensation. When Aurora B, the kinase of H3S10ph, was inhibited, the pSer22 modification on lamin A still happened, although the mitosis period was shortened, which indicated that phosphorylation on lamin A was relatively independent from Aurora B.

## Materials and Methods

### Plasmid construction

pSer22 lamin A FRET biosensor consists of 5 parts: FHA2 domain, yellow fluorescent protein YPet, linker [Eevee (EV) linker or 34-mer], enhanced cyan fluorescent protein (ECFP), and full-length lamin A (or lamin A nanobody [20]). A flexible linker was used to separate FRET pair YPet/ECFP. We amplified encoding sequences of each fragment from our previously reported plasmids [21] or cDNA of HeLa cells and then cloned them into a pSin vector backbone by Gibson assembly (NEB, E2621S). To further examine the specificity of the biosensor, FHA2 mutated biosensor R605A and lamin A mutant biosensors S22A/S392A were constructed. A point mismatch on primer sequence was introduced into the coding sequence of FHA2 or lamin A by polymerase chain reaction (PCR) and then cloned to pSin backbone by Gibson assembly. The final constructs were verified by Sanger sequencing at Genewiz. All the primers used in this study were listed in Table S1.

### Cell culture and transfection

HeLa cells were used for biosensor evaluation and analysis in this study. The cells were cultured in Dulbecco’s modified Eagle’s medium (DMEM) that contained 10% fetal bovine serum (FBS; Sigma-Aldrich) and 1% penicillin/streptomycin (Gibco). After that, the cells were grown in a humidified incubator with 5% CO_2_ at 37°C. For transfection of biosensors, HeLa cells were seeded in a 24-well plate for 24 h and then transfected with 200 ng of plasmid by Lipofectamine 3000 Transfection Reagent (Invitrogen) according to the manufacturer’s instruction.

### Drug treatments

The hypotonic condition was generated by using DMEM culture medium containing 50% water. To get G_1_-S boundary cells, 2 mM thymidine (Sigma, T1895) was added for 18 h in HeLa cells. After washing by 1× phosphate-buffered saline (PBS) twice, cells were then released into regular medium for 8 h and then treated with 2 mM thymidine for another 18 h. To arrest cells at G_2_-M transition, RO-3306 (Beyotime, SC6673-5mg) was added to HeLa cells at a final concentration of 9 μM for 18 h. To inhibit Aurora B activity, hesperadin (MCE, HY-12054) was added to HeLa cells at a final concentration of 200 nM. Hesperadin was added 0.5 h before the imaging with RO-3306 in the medium. When FRET imaging started, cells were released from RO-3306 but with hesperadin treatment during the imaging process.

### Western blot

LAPS wild-type (WT) and mutants were transfected into HeLa cells in a 12-well plate using Lipofectamine 3000 (Invitrogen, L3000015) according to the manufacturer’s protocol. After expression for 48 h, plain HeLa and transfected HeLa cells were harvested and lysed in radioimmunoprecipitation assay (RIPA) buffer (Thermo, 89900). Cell lysis was centrifugated at 15,000*g* for 15 min at 4°C, and the supernatant was collected for Western blot (WB) analysis. Each sample was stained with 3 primary antibodies: lamin A [Cell Signaling Technology (CST), 4777S], pSer22 lamin A (CST, 13448S), and green fluorescent protein (GFP) (CST, 2956S). The blots were stripped off by stripping buffer (Thermo, 21059) at 37°C for 1 h before the next round of antibody staining.

### Immunofluorescence staining

Cells were seeded on the glass-bottom dishes for 12 to 24 h, then rinsed with 1× PBS for 3 times, and fixed in 4% (w/v) paraformaldehyde for 30 min. The fixed cells were further rinsed with 1× PBS for 3 times, permeabilized in 0.25% (v/v) Triton X-100 for 10 min, and washed with 1× PBS for 3 times. Subsequently, samples were blocked with 5% bovine serum albumin (BSA) for 2 h at room temperature. H3S10ph antibody (1:500; CST, 9706S) and pSer22 lamin A antibody (1:500; CST, 13448) were incubated overnight at 4°C, and cells was rinsed with 1× PBST (1× PBS with 0.1% Tween 20). Next, the samples were incubated with secondary antibody at room temperature for 2 h, e.g., goat anti-mouse Alexa Fluor 488 (1:1,000; Abcam, ab150077) and Alexa Fluor 647 (1:1,000; CST, 4410S). Finally, samples were rinsed with 1× PBST and incubated with Hoechst 33342 (1:2,000, CST, 4082S) for 10 min at room temperature for nuclei staining. After washing by 1× PBST, the samples were mounted with VECTASHIELD Antifade Mounting Medium (Vectorlabs, H-1000-10) and imaged by Dragonfly Spinning Disk Confocal Microscopy System (Dragonfly CR-DFLY-202-40, USA).

### FRET imaging and analysis

After 48 h of transfection, cells expressing WT or mutant LAPS were seeded on the glass-bottom dishes (Biosharp, BS-15-GJM). Before imaging, the cell culture was switched to DMEM without phenol red. FRET imaging was conducted on a Dragonfly Confocal Microscopy System equipped with a 7-laser, electron multiplying charge-coupled devices (EMCCD) camera and live-cell workstation. The ECFP and FRET channels were excited by a 445-nm laser. The 478- and 571-nm filters were used to collect the emission of ECFP and FRET, respectively. The ECFP and FRET images were processed and quantified by the image analysis software package Fluocell (http://github.com/lu6007/fluocell), and the ECFP/FRET ratio was calculated and analyzed by GraphPad Prism software.

### CRISPR-mediated LMNA gene knockout

To knock out LMNA genes, we first inserted LMNA guide RNA (gRNA) into pSpCas9(BB)-2A-GFP (PX458) (Addgene, #48138). Herein, we designed primers for 2 gRNAs (gRNA1: 5′-GCGAGCTCCATGACCTGCGG-3′, gRNA2: 5′-TCTCAGTGAGAAGCGCACAT-3′) and the vectors were assembled by Golden Gate assay. Plasmids were verified by Sanger sequencing. Then, 0.4 million C2C12 cells were seeded into a 6-well plate the day before transfection. LMNA-PX458-gRNA1 (1.25 μg) and LMNA-PX458-gRNA2 (1.25 μg) were cotransfected into C2C12 with Lipofectamine 3000 Reagent (Invitrogen, L3000015). Forty-eight hours after transfection, cells were subjected to fluorescence-activated cell sorting (FACS) (CytoFLEX SRT, Beckman) to isolate GFP-positive monoclonal cell into 96-well plates. The knockout efficiency was further verified by genotyping PCR with LMNA-specific primers (LMNA-F: 5′-TCGAGGCT-CTTCTCAACTCC-3′, LMNA-R: 5′-AGGTGAGCAGGCAAAT-GG-3′) as well as Sanger sequencing.

### Statistical analysis

All experiments were conducted in triplicate and repeated at least twice unless otherwise stated. All the data were presented as mean ± standard error of the mean (SEM). The significance of differences was analyzed with one-way analysis of variance (ANOVA) followed by Tukey’s honest significant difference test using GraphPad Prism software (version 8.0, GraphPad Software Inc.). Significant differences were determined by *P* values (*P < 0.05, **P < 0.01, ***P < 0.001).

## Results

### LAPS design and characterization

**Fig. 1.**
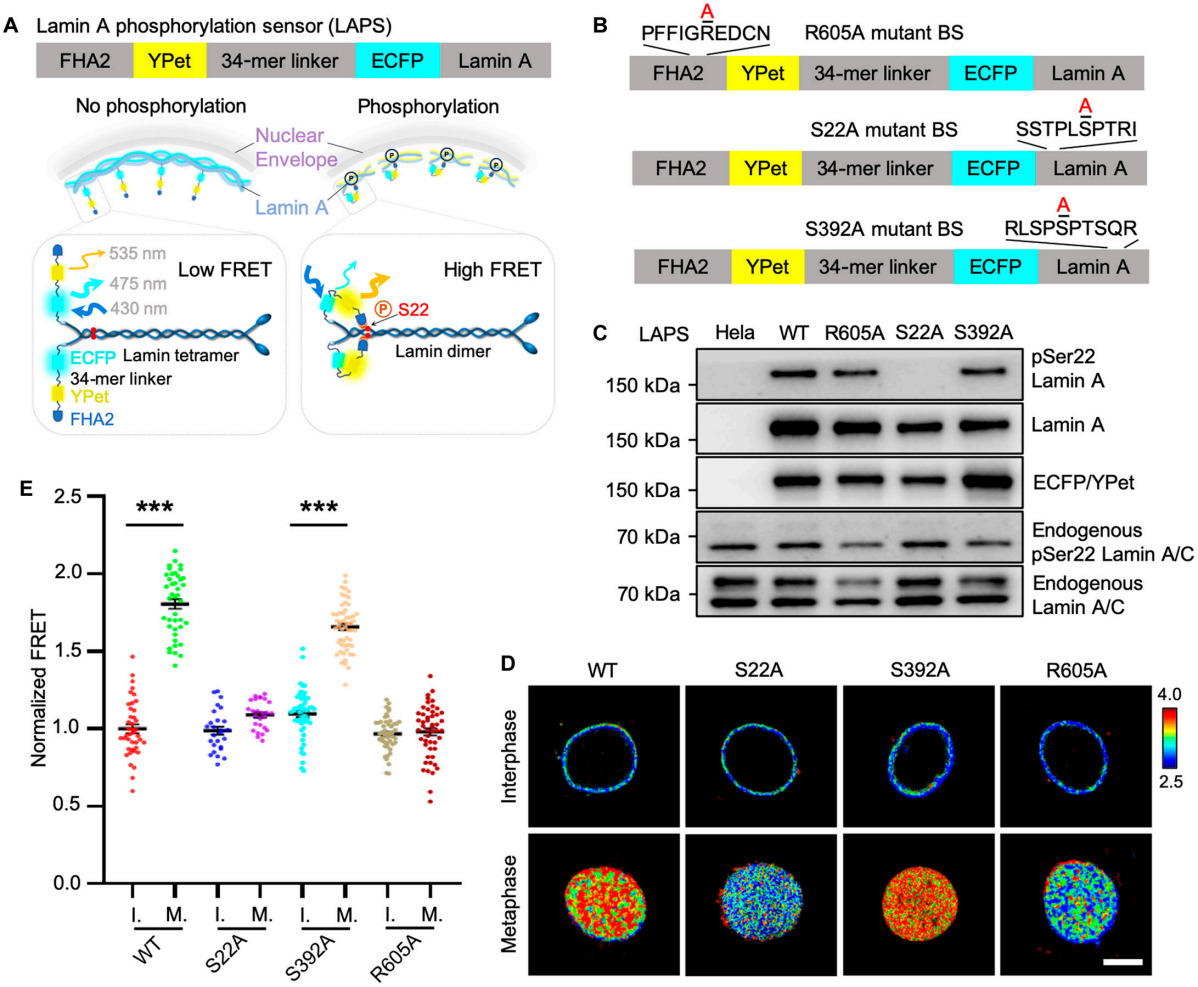
Development and characterization of LAPS. (A) Schematic diagram of LAPS. At the resting state when lamin A is not phosphorylated, the excitation of ECFP could not trigger a FRET event. When lamin A gets phosphorylated, the FHA2 domain can sense the phosphorylation site, which leads to a tremendous conformational change and proximity of the FRET pair protein. An increase in the FRET ratio could then be observed. Thus, high FRET corresponds to the phosphorylation state of lamin A, which mediates depolymerization of lamin A protein. (B) Construct of WT LAPS and 3 mutants of LAPS. R605A mutation occurs on the FHA2 domain, while S22A and S392A are on lamin A. (C) Expression and phosphorylation of LAPS in HeLa cells. All the groups (left to right) represent plain HeLa cells and HeLa cells expressing WT LAPS, R605A LAPS, S22A LAPS, and S392A LAPS. Bands with larger size (~170 kDa) refer to constitutive expression of biosensors. ECFP/YPet band is detected by GFP antibody to indicate the size of biosensor itself. (D) Representative images of the FRET ratio from HeLa cells expressing WT, S22A, S392A, and R605A LAPS in interphase and mitosis. Scale bar, 10 μm. (E) Quantitative analysis of the FRET ratio from (D) (*n* > 20). I, interphase; M, mitosis. ***P < 0.001. All the FRET ratio was normalized to the corresponding interphase group. Briefly, the FRET ratio from the nuclear envelop region was calculated for individual cells in each interphase group, and the FRET ratio from the entire nuclei was calculated for individual cells in each mitosis group. Then, the average FRET ratio was calculated with more than 20 cells for each interphase group. Then, all the FRET ratio was normalized to the average FRET ratio of the corresponding interphase group.

To obtain highly sensitive and specific FRET biosensor for pSer22 lamin A detection, we linked the lamin A nanobody and FHA2 domain with 2 fluorescent proteins ECFP and YPet in the middle. A flexible linker (EV linker) with 120 amino acids was inserted between ECFP and YPet according to our previous work [21]. This biosensor was named as pLamin A biosensor 1.0 (Fig. S1A). Different levels of pSer22 on lamin A in interphase cells and mitotic cells were observed by pLamin A biosensor 1.0 with approximately 10% dynamic changes (Fig. S1B and C), indicating the low sensitivity of pLamin A biosensor 1.0. Considering that the nanobody targeting site is the end of the coiled-coil domain of lamin A, so it is far away from the pSer22 site [20]. To increase the specificity to pSer22, we switched the substrate into full-length lamin A based on pLamin A biosensor 1.0, which is called pLamin A biosensor 2.0 (Fig. S2A). Our results showed that the NE location of the biosensor was dramatically improved (Fig. S2B). The WT biosensor successfully tracked the dynamic changes of lamin A dephosphorylation when cells exited mitosis, while R605A mutation in FHA2 impaired the mitotic response (Fig. S2B to H). However, the fold change of FRET ratio between mitosis and interphase was still unsatisfied (Fig. S2). Considering that the FHA2 domain had low affinity with phosphorylated serine [22], we shortened the EV linker from 120 amino acids to 34 amino acids to enhance the possibility of FHA2 association with phosphorylated serine and named it as LAPS (Fig. 1A).

To comprehensively verify the specificity and sensitivity of LAPS, we designed distinct mutations on LAPS: one was serine 22 to alanine at the N terminus of the lamin A region on LAPS, the other was serine 392 to alanine at the C terminus of the lamin A region on LAPS, and another was arginine 605 to alanine in the FHA2 domain on LAPS to have R605A mutant biosensor with disabled recognition ability of phosphorylated serine (Fig. 1B). Upon transient transfection in HeLa cells, we detected lamin A and its phosphorylation level on both biosensors (170 kDa) and endogenous lamin A (70 kDa) by WB, and 170-kDa band of pSer22 lamin A disappeared in the S22A group but not in the S392A group, indicating that LAPS behaved as endogenous lamin A as we designed (Fig. 1C). The live-cell imaging results showed the correct location of all the biosensors around the NE, which indicated the correct expression and function of WT and mutated biosensors in mammalian cells (Fig. 1D). There were similar basal level FRET ratios among WT and mutant biosensors in interphase cells because of little pSer22 modification in the interphase, but much higher FRET ratios in the mitotic cells expressing WT and S392A biosensors due to the high pSer22 level during mitosis. However, the apparent change of FRET ratio between interphase and mitosis was abolished by S22A and R605A mutant because of the incapable phosphorylation on the S22A site and the incapable recognition of phosphorylation by FHA2 domain with R605A mutation (Fig. 1D and E), implying that LAPS detected pSer22 specifically. In addition, the dynamic range of LAPS was around 80% changes in terms of FRET ratio (Fig. 1E), which was increased drastically compared to pLamin A biosensor 1.0 and 2.0. Therefore, all the results demonstrated the high specificity and sensitivity of LAPS to pSer22 modification on lamin A.

### LAPS detects pSer22 dynamics in cell adhesion and hypotonic condition

**Fig. 2.**
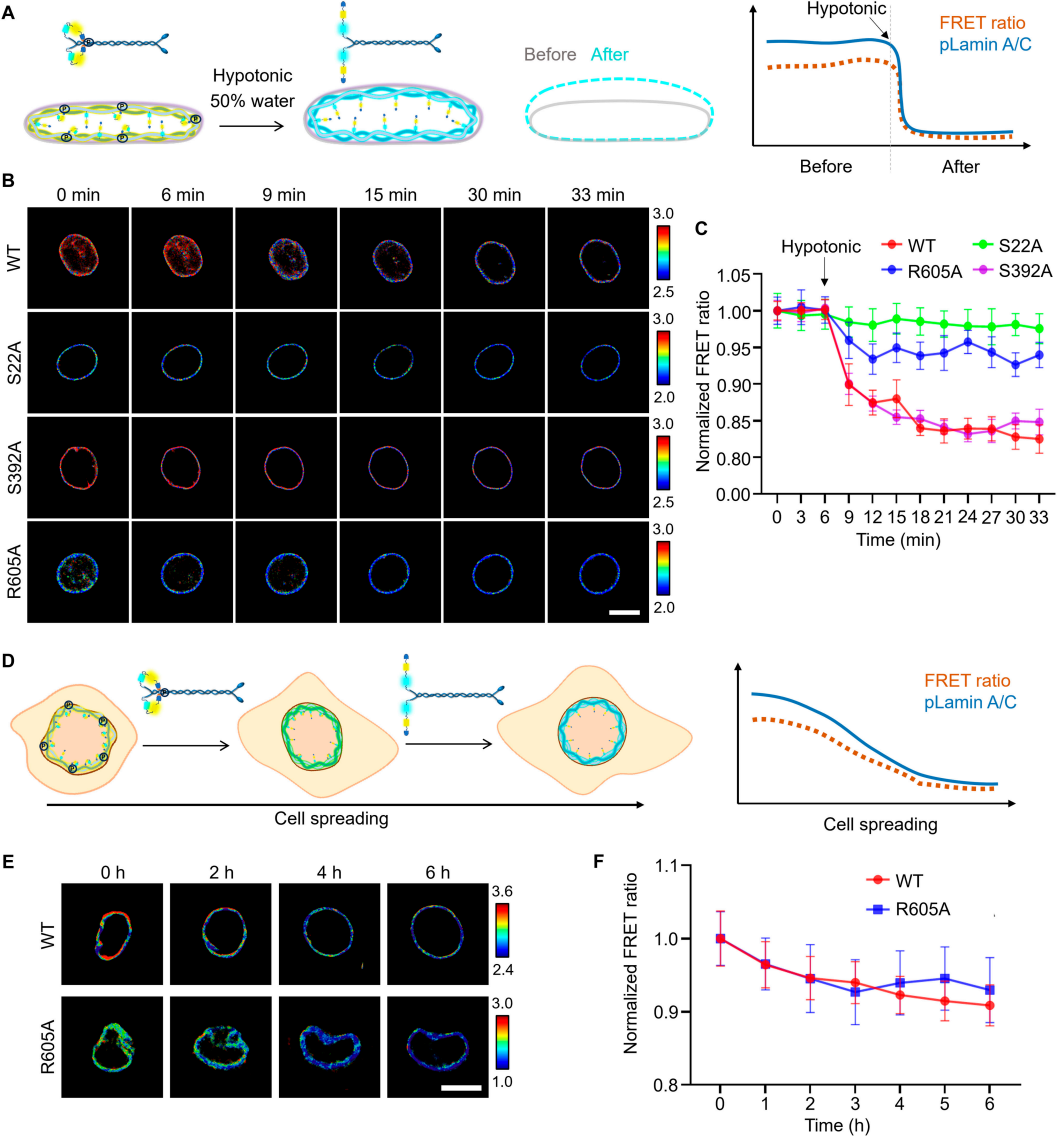
The effect of nuclear deformation on lamin A phosphorylation. (A) The diagram shows the change in lamin A phosphorylation at low osmolality. The hypotonic condition was treated by using DMEM culture medium containing 50% water, creating an immediate low osmolality environment in which the nuclei swelled and increased the pressure, mediating higher levels of dephosphorylation of lamin A, opening of the FRET biosensor structure, and decreasing the FRET ratio. (B) Time-lapse FRET/ECFP ratio images of the WT LAPS and the other 3 mutants in HeLa cells treated with hypotonic condition for about half an hour. The color bar indicates high (red) and low (blue) FRET ratio. Scale bar, 10 μm. (C) Time courses of the normalized FRET/ECFP ratio of the WT and mutants LAPS (*n* = 20) in HeLa cells treated with H_2_O. All the FRET ratios at different time points were normalized to the basal FRET ratio at time point zero. (D) The schematic diagram shows the dynamic process of lamin A phosphorylation during cell spreading. The detached cells are with higher levels of lamin A dephosphorylation, opening of the FRET biosensor structure and decreasing the FRET ratio, and vice versa. (E) Time-lapse FRET/ECFP ratio images of the WT LAPS and R605A mutant during HeLa cell adhesion onto the matrix. The color bar indicates high (red) and low (blue) levels of pSer22 lamin A. Scale bar = 10 μm. (F) Time courses of the normalized FRET ratio from (E) (*n* = 17). All the FRET ratios at different time points were normalized to the basal FRET ratio at time point zero.

It has been shown that the phosphorylation of lamin A changes under mechanical force variation [23]. To visualize the dynamics of pSer22 on lamin A, we transiently transfected HeLa cells with WT LAPS and 3 mutants of LAPS (Fig. 2A to C). Under the hypotonic conditions, we observed the gradual reduction of FRET ratio in the WT group, which was much lower than S22A and R605A groups, indicating that pSer22 on lamin A decreased during the increase of cell tension. Meanwhile, the S392A group changed similarly to the WT group, further implying that our biosensors mainly detected the phosphorylation on Ser22 as designed. WB results further proved the reduction of pSer22 modification on endogenous lamin A and LAPS (Fig. S3). The other process monitored by LAPS was cell adhesion. Literatures showed that lamin A phosphorylation decreased progressively during cell adhesion [15], which was also observed by our WT LAPS, whereas the R605A mutant showed relatively low response (Fig. 2D to F). Putting together, LAPS demonstrated the dynamic changes of pSer22 lamin A in living cells under extracellular mechanical changes, which plays important roles on the regulation of dynamic polymerization and depolymerization of lamina network for mechanical adaptation.

### LAPS detects pSer22 dynamics in cell cycle

**Fig. 3.**
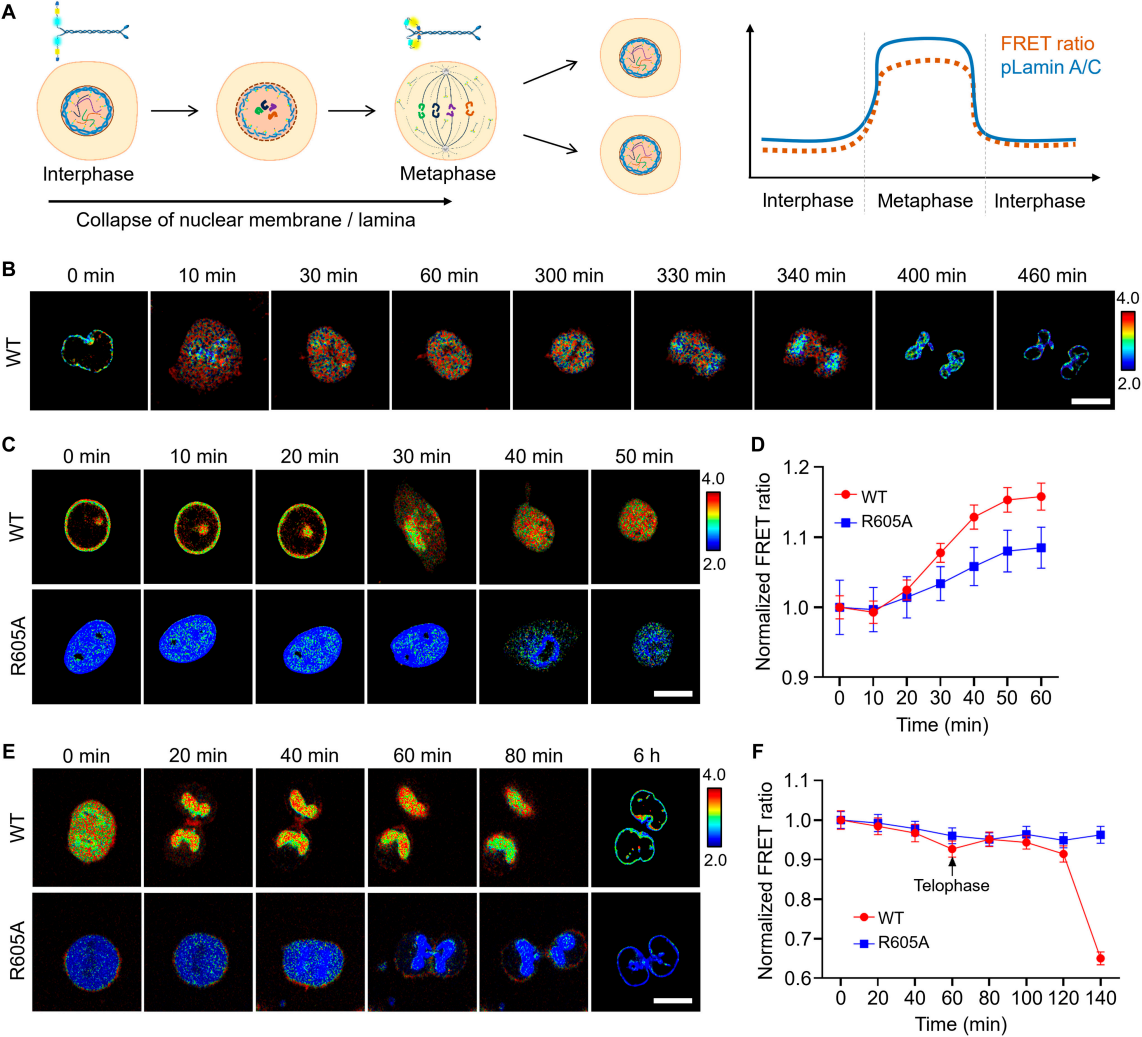
The dynamics of pSer22 on lamin A were monitored by LAPS during cell cycle. (A) In the process of mitosis, the nuclear membrane undergoes breakdown/reforming cycles and lamin A undergoes depolymerization and polymerization cycles, in which pSer22 plays critical role, resulting in the increase and decrease of the FRET ratio in principle. (B) Time-lapse FRET ratio images in HeLa cells expressing WT LAPS during cell mitosis upon RO-3306 release at *t* = 0. (C) Time-lapse FRET ratio images of the WT and R605A LAPS in HeLa cells as the cell progressed from interphase into mitosis. HeLa cells were released from double thymidine in this experiment. Scale bar, 10 μm. (D) Time courses of the normalized FRET ratio of the WT and R605A LAPS (*n* = 20, 20) in HeLa cells as the cell progressed from interphase into mitosis. All the FRET ratios at different time points were normalized to the basal FRET ratio at time point zero. (E) Time-lapse FRET ratio images of the WT and R605A LAPS in HeLa cells as the cell progressed from mitosis into interphase. Scale bar=10 μm. (F) Time courses of the normalized FRET ratio of WT and R605A (*n* = 20, 20) in HeLa cells as the cell exited mitosis. All the FRET ratios at different time points were normalized to the basal FRET ratio at time point zero.

LAPS detected the significant pSer22 modification in mitotic cells compared to interphase cells (Fig. 1D and E), so we wondered the entire dynamics of pSer22 on lamin A during cell cycle. We first synchronized the HeLa cells transiently expressing WT LAPS by CDK1 inhibitor RO-3306 to late G_2_ phase [24] and then released the cells to go through the whole mitosis (Fig. 3A). We found that the NE broke down immediately after removal of RO-3306. The FRET ratio extensively raised as the NE breakdown occurred (Fig. 3B and Movie S1). In the telophase, the FRET ratio on the chromosome was reduced, which was consistent with the previous reports that pSer22-modified lamin A was dephosphorylated by the phosphatase PP1 that was located on the chromosome surface in the telophase (Fig. 3B) [25]. The time-course images exhibited the high spatiotemporal resolution of LAPS. To further refine the observation of the changes in pSer22 modification on lamin A during the whole cell cycle, we did more careful quantification of the FRET ratio from the interphase released from double thymidine to mitosis or from mitosis to interphase. As the cell progressed from interphase into mitosis, the NE gradually broke down and the FRET signal increased (Fig. 3C and D). When the mitotic cells entered the interphase, the evenly distributed fluorescent signal was gradually concentrated in two regions within the cell representing the reassembled nuclei. The FRET signal was declined along cell division (Fig. 3E and F). The decrease in the FRET ratio reflected the reduction of pSer22 modification on lamin A, which occured when lamin A concentrated around the chromosome in nascent nucleus to prepare for reconstruction of nuclear membrane. On the other hand, cells expressing the mutant LAPS R605A lacked the FRET signaling response during mitosis.

**Fig. 4.**
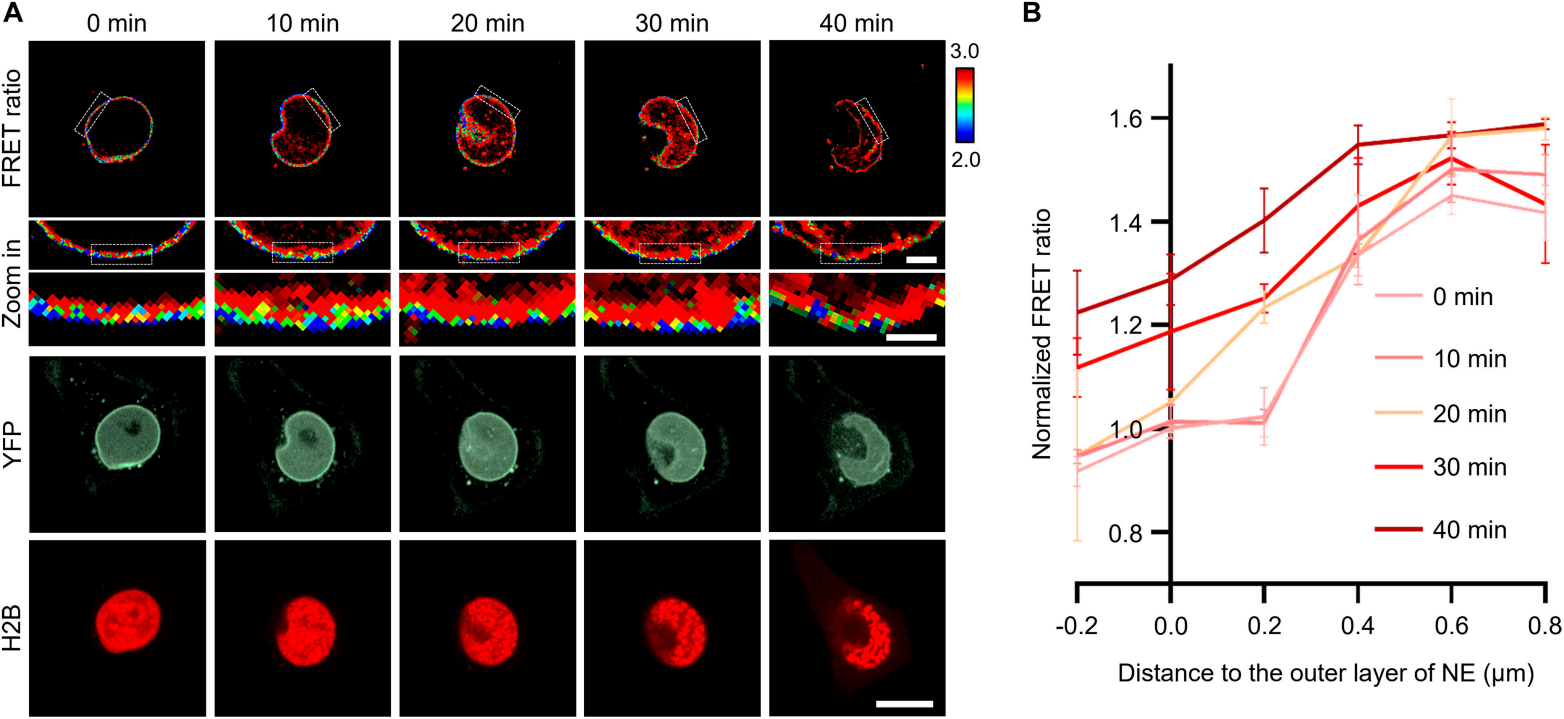
The dynamic and spatial feature of pSer22 lamin A at mitosis entry. (A) Time-lapse imaging was performed in HeLa cells expressing LAPS and H2B-RFP (red fluorescent protein) released from RO-3306. FRET ratio: Time-lapse FRET ratio images. Zoom in: Enlarged FRET ratio images. YFP (yellow fluorescent protein): YFP only channel to show the location of LAPS. H2B: H2B-RFP to indicate chromatin. Scale bar, 10 μm. (B) Time courses of the normalized FRET ratio in HeLa cells in (A). The abscissa 0 indicated the outer layer of the nuclear membrane. *n* = 20. The FRET ratio increased nearby 0 μm when cells got into mitosis, indicating the propagation of pSer22. The FRET ratio was normalized to data at 0 min and 0 μm.

To further investigate the pSer22 propagation at the nuclear periphery in spatial resolution, we precisely tracked this process with LAPS on confocal microscopy. The results showed that the pSer22 modification on lamin A occurred first inside the nuclear membrane, as the phosphorylated portion of lamin A transported into the nucleoplasm. As mitosis proceeded, lamin A in the immediate vicinity of the inner nuclear membrane was phosphorylated until the nuclear membrane completely collapsed (Fig. 4A). We profiled the FRET ratio from the inside to outer layer of NE, and obviously found that the FRET ratio at the outer layer of NE was as high as that at the inside layer when chromatin got condensed into chromosomes, which took about half an hour to spread out on the NE since cells got released from RO-3306 (Fig. 4A and B), indicating that pSer22 on lamin A occurred inside the nuclear membrane first to the outer layer of NE later.

### A positive correlation between H3S10ph and pSer22 Lamin A at mitosis entry

**Fig. 5.**
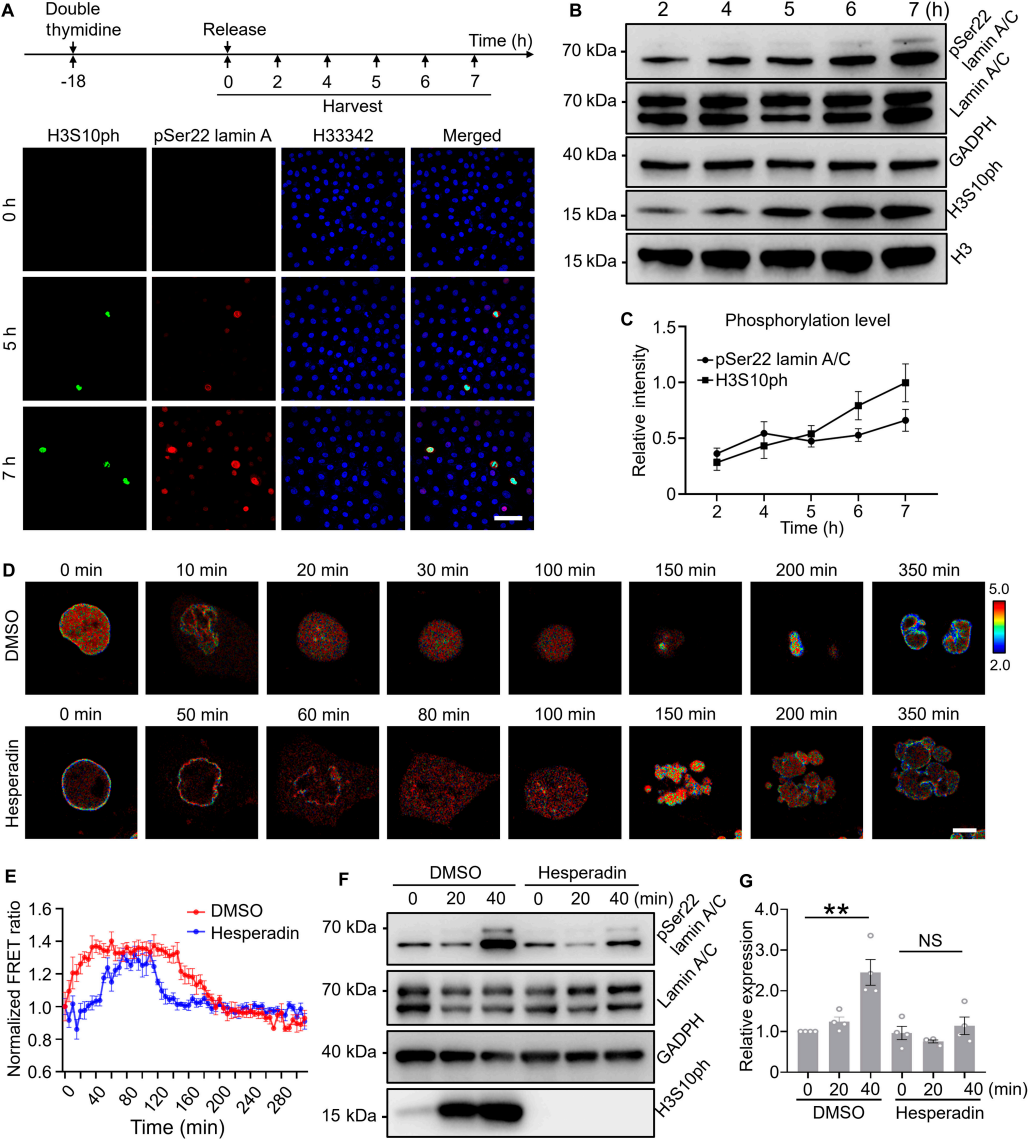
A positive correlation between H3S10ph and pSer22 lamin A at mitosis entry. (A) Immunofluorescence was used to detect the changes of H3S10ph and pSer22 lamin A in HeLa cells from early S phase to M phase after double thymidine release. Green represents H3S10ph, red represents pSer22 lamin A, and blue represents nucleus. Cells were released after double thymidine synchronizaction to the G_1_-S boundary, and samples were collected at different time points after release. Scale bar, 100 μm. (B) WB was used to detect the changes of H3S10ph and pSer22 lamin A in HeLa cells from early S phase to M phase, and the cells were synchronized to G_1_-S boundary after double thymidine treatments and then released at different time points. (C) WB quantification from (B). WB intensity was normalized to respective time point 7 h data. (D) Cells were pretreated with RO-3306 and synchronized to G_2_-M transition. Time-lapse imaging of WT LAPS in HeLa cells treated with dimethyl sulfoxide (DMSO) or 200 nM hesperadin. Scale bar, 10 μm. (E) Time courses of the normalized FRET ratio of the HeLa cells treated with DMSO or 200 nM hesperadin in (D) (*n* = 17). The FRET ratio was normalized to respective time point zero data. (F) WB was used to detect the changes of H3S10ph and pSer22 lamin A when cells entered mitosis with either DMSO or hesperidin treatment. The cells were synchronized to the G_2_-M transition after RO-3306 treatment and then collected at 0, 20, and 40 min for WB. (G) Quantified bar graph for (F). **P < 0.01, not significant (NS); *n* = 4.

Mitosis is accompanied by a significant increase in both lamin A phosphorylation and H3S10 phosphorylation (H3S10ph). We are curious about which phosphorylation event occurs first and if they have a crosstalk with each other. To address this, we costained pSer22 lamin A and H3S10ph in unsynchronized HeLa cells and did observe that pSer22 lamin A and H3S10ph intensity increased dramatically in the mitosis. Interestingly, there was a small portion of H3S10ph in a confined region near the nuclear membrane in some population of cells with a moderate level of lamin A phosphorylation (Fig. S4). To precisely explore the spatiotemporal relationship between pSer22 lamin A and H3S10ph during the cell cycle progression, we synchronized the HeLa cells at G_1_-S boundary by double thymidine arrest. The immunostaining and WB results demonstrated that pSer22 lamin A and H3S10ph gradually increased upon release (Fig. 5A to C). However, pSer22 lamin A showed a slight raise in the S phase, which might be governed by cell cycle-dependent kinases in the S phase. Compared with pSer22 lamin A, the H3S10ph was a more specific event in mitosis. Then, we explored the H3S10ph level during mitosis in lamin A knockout cells. The immunostaining and WB results proved that lamin A loss had no effect on mitosis-induced H3S10ph (Fig. S5). To further uncover the crosstalk between pSer22 lamin A and H3S10ph, we inhibited the Aurora B kinase activity by hesperadin [26,27] and tracked the pSer22 lamin A dynamics by our LAPS biosensor (Fig. 5D and E). The results indicated that the mitosis process was delayed and shortened after hesperadin treatment as reported [28]. Meanwhile, pSer22 on lamin A was also delayed in the hesperadin-treated group, but the mitosis-induced FRET raise was maintained, which implied that Aurora B indeed governed cell mitosis entry, but it was not the key kinase of pSer22 lamin A. To further explore the delay of mitosis induced by hesperadin, we synchronized the HeLa cells at G_2_-M boundary by RO-3306 and inhibited the Aurora B activity by hesperadin. The results of WB indicated that phosphorylation of H3S10 was completely abolished and pSer22 modification on lamin A was prevented, which should be the consequence of delayed mitosis (Fig. 5F and G), suggesting the positive correlation between H3S10ph and pSer22 lamin A at mitosis entry.

## Discussion

In summary, we developed a genetically encoded FRET biosensor LAPS for live-cell imaging of pSer22 lamin A with high specificity and sensitivity. Utilizing LAPS, we uncovered the gradual reduction of pSer22 modification on lamin A during cell adhesion and hypotonic treatment, which demonstrated how nuclear deformation and cell tension regulated pSer22 lamin A. In addition, we monitored the dynamic changes of pSer22 lamin A during mitosis with high spatiotemporal resolution, consistent with previous reports that lamin A was extensively pSer22 modified during mitosis [8,9,29–32]. We found that lamin A was phosphorylated from the interior of nuclear membrane to the boundary and was dephosphorylated as the cells exited mitosis. Considering the synchronicity of pSer22 lamin A and H3S10ph, we focused on the crosstalk of these 2 phosphorylation events. Knockout lamin A did not affect H3S10ph in cell mitosis. Inhibition of Aurora B, the kinase of H3S10ph, delayed and shortened the mitotic process, while pSer22 modification on lamin A was maintained.

We observed the positive correlation between H3S10ph and pSer22 lamin A at mitosis entry (Fig. 5). The enzyme of H3S10ph in mitosis is Aurora family kinases, especially Aurora B [33]. The kinase responsible for pSer22 lamin A in mitosis is CDK1, which controls the entry of mitosis [34]. Previous studies reported that Aurora B function in mitosis can be regulated by CDK1 [35], so we speculated that CDK1 might play an important role in regulating this positive correlation between H3S10ph and Lamin A pSer22 at mitosis entry. With *LMNA* knockout, we tried to remove all lamin A and pSer22 lamin A and investigated the causative relationship to H3S10ph, but did not see any effect on H3S10ph, indicating that lamin A and pSer22 lamin A are not regulating H3S10ph directly; instead, their kinases may have crosstalk.

We also observed H3S10ph puncta in very early prometaphase accompanied with moderate pSer22 lamin A, which implied that pSer10 modification of histone 3 may start at the nuclear membrane, which eventually prorogated onto the entire chromosome. However, so far, we did not work out the simultaneous monitoring of H3S10ph and pSer22 lamin A by dual FRET imaging due to the low sensitivity of H3S10ph biosensor, which was reconstructed with mOrange/Fusion red FRET pair according to previous work [21]. So, a new red FRET pair with high efficiency should be developed in the near future. Moreover, faster lamin A turnover on soft gels was found, which could be one of important mechanism on how heterochromatin disassociation was regulated at the nuclear periphery [15]. To track the dynamic changes of pSer22 lamin A in diverse mechano-chemical environments, we need to optimize the sensitivity of LAPS further. The FHA2 domain recognizes the phosphorylated threonine in the native circumstance [22]. The affinity of FHA2 domain to phosphorylated serine was relatively low, which impaired the binding efficiency of LAPS. Therefore, novel FHA2 mutants with higher binding affinity to pSer are needed to obtain more dynamic changes of LAPS, which can be optimized by directed evolution of FHA2. In addition, introducing LAPS into cells could increase the lamin A level in the transfected cells, which might affect the cellular function monitored by LAPS, so it would be better to examine the cell function first to make sure that the increase of lamin A will not affect cell function before live-cell imaging by LAPS. Otherwise, using CRISPR knock-in to replace endogenous *LMNA* gene with LAPS will be recommended.

## Data Availability

The data that support the findings of this study are available from the corresponding authors upon reasonable request.
