# Supplementary material for: Genetically Encoded Fluorescence Resonance Energy Transfer Biosensor for Live-Cell Visualization of Lamin A Phosphorylation at Serine 22
Source: Biomater Res. 2024 Oct 22;28:0091. doi: 10.34133/bmr.0091 (PMC11494032; doi:10.34133/bmr.0091)
Supplement: Supplementary 1 — Figs. S1 to S5 Table S1 Movie S1 [file bmr.0091.f1.zip › clean SI version-final.doc]

**Front Matter**

Title

Genetically Encoded FRET Biosensor for Live-cell Visualization of Lamin A Phosphorylation at Serine 22

**Authors**

Jian Liu^1,†^, Qianqian Li^1,†^, Jinfeng Wang^1,†^, Juhui Qiu^2,*^, Jing Zhou^3,*^, Qin Peng^1, *^

**Affiliations**

^1^ Shenzhen Bay Laboratory, Shenzhen, 518132, China.

^2^ Key Laboratory for Biorheological Science and Technology of Ministry of Education, State and Local Joint Engineering Laboratory for Vascular Implants, College of Bioengineering, Chongqing University, Chongqing, 400030, China.

^3^ Department of Physiology and Pathophysiology, School of Basic Medical Sciences, State Key Laboratory of Vascular Homeostasis and Remodeling, Department of Cardiology, Peking University Third Hospital, National Health Commission Key Laboratory of Cardiovascular Molecular Biology and Regulatory Peptides, Beijing Key Laboratory of Cardiovascular Receptors Research, Peking University, Beijing, 100191, China.

^†^ These authors contributed equally

Corresponding author: Qin Peng (pengqin@szbl.ac.cn), Jing Zhou (jzhou@bjmu.edu.cn), Juhui Qiu (jhqiu@cqu.edu.cn)

Supplementary Materials

Fig S1-S5

Table S1

Movie S1

**Fig S1.** pLaminA biosensor 1.0. **a.** Schematic diagram of the pLaminA FRET biosensor 1.0. A flexible linker (EV linker) with 120 aa was inserted between ECFP and YPet. At resting state when lamin A is not phosphorylated, the excitation of ECFP cannot trigger a FRET event. When lamin A gets phosphorylated, the FHA2 domain can bind to the phosphorylation site, which leads to a tremendous conformational change and proximity of the FRET pair protein. An increase in the FRET ratio could be then observed. **b.** Quantitative results of FRET ratio in HeLa cells expressing pLaminA biosensor 1.0 in interphase and mitotic (M) phase (n = 29, 24). **c.** The time-lapse FRET ratio images of one representative HeLa cells expressing pLaminA biosensor 1.0 in cell mitosis. Scale bar: 10 μm.

**Fig S2.** pLaminA biosensor 2.0. **a.** Schematic diagram of the pLaminA FRET biosensor 2.0. A flexible linker (EV linker) with 120 aa was inserted between ECFP and YPet. The lamin A protein is connected behind the ECFP. At resting state when lamin A is not phosphorylated, the excitation of ECFP cannot trigger a FRET event. When lamin A gets phosphorylated, the FHA2 domain can bind to the phosphorylation site, which leads to a tremendous conformational change and proximity of the FRET pair protein. An increase in the FRET ratio could be then observed. **b.** In the process of mitosis, the nuclear membrane undergoes breakdown/reforming cycles and lamin A undergoes depolymerization and polymerization cycles, in which pSer22 plays critical role, resulting in the increase and decrease of FRET ratio in principle. **c and f.** Time-lapse FRET/ECFP ratio images of the pLamin 2.0 biosensor WT and R605A in HeLa cells as the cell progressed from mitosis into interphase. Scale bar=10 μm. **d and e.** Quantitative results of FRET ratio in HeLa cells from c. g and h. Quantitative results of FRET ratio in HeLa cells from **f**.

**Fig S3.** Hypotonic conditions induced the reduction of pSer22 level on Lamin A/C. **a.** WB of lamin A/C, pSer lamin A/C, GAPDH in HeLa cells overexpressing WT and mutant LAPS after hypotonic treatment. **b**. pSer22 level related to total LAPS in HeLa cells overexpressing WT and mutant LAPS after hypotonic treatment. n=3, WT: ****p* <0.001, S22A: not applicable (N/A), S392A: **p* <0.05, R605A: ****p* <0.001. **c**. The relative pSer22 level normalized to the total endogenous lamin A/C protein in HeLa cells overexpressing WT and mutant LAPS after hypotonic treatment. n=3, WT: ***p* <0.01, S22A: ****p* <0.001, S392A: ****p* <0.001, R605A: ****p* <0.001.

**Fig S4.** Immunofluorescence images to show H3S10ph and pSer22 lamin A levels in different view of HeLa cells from G2 to M phase. Green represents H3S10ph, red represents pSer22 lamin A, and blue represents nucleus, scale bar = 10 μm.

**Fig S5.** Knockout LMNA had no effect on H3S10ph. **a.** Immunofluorescence was used to detect H3S10ph and pSer 22 lamin A in WT and LMNA knockout C2C12 cell lines. Green represents H3S10ph, red represents pSer 22 lamin A, and blue represents nucleus, scale bar = 50 μm. **b.** WB detected the changes of H3S10ph and pSer 22 lamin A in WT and *LMNA* knockout cell lines from G2 to M phase. The cells were synchronized to the G2/M phase after RO-3306 treatment, and the cells were collected at 0, 20, and 40 min for WB detection. **c and d.** WB quantification of both phosphorylation changes in WT cells (c) and LMNA KO cells (d) from b. (n=3)

**Table S1.** Primers for cloning used in this study

| **Constructs** | **Primers** | **Primer sequences** |
| --- | --- | --- |
| **pLamin A biosensor 1.0** | FHA2 F1 | GGAGTTCGTGACCGCCGCCACCAAGGGTAATGGTAGGTT |
|  | FHA2 R1 | TATGATCTAGAGTCGCGGCCGCCTACACCTTGCGTTTTTTCTTGGG |
|  | YPet F1 | AATTCTGCAGTCGACGGTACCGCATGTCTAAAGGTGAAGAATTAT |
|  | YPet R1 | ATCCGCCTGCGCTAGACCCAGCAGCAGTCAAAAATTC |
|  | ECFP F1 | GGGTCTAGCGCAGGCGGAT |
|  | ECFP R1 | GGCGGCGGTCACGAACTCC |
| **pLamin A biosensor 2.0** | FHA2 F2 | GAATTGATCCTTCGAAGCTAGCGCCACCATGAAGGGTAATGGTAGGTTTTTA |
|  | FHA2 R2 | AAGCTTGATATCGAATTCCTTAACTTTTTCACCAAATCTTT |
|  | YPet F2 | AGGAATTCGATATCAAGCTT |
|  | ECFP R2 | GGCGGCGGTCACGAACTCCA |
|  | Lamin A F | TGGAGTTCGTGACCGCCGCCGGGGGAAGCGGCGGGACCAT |
|  | Lamin A R | TCGAGCTCAAGCTTCGAATTCCTACATGATGCTGCAGTTCTGGG |
| **LAPS** | 34-mer F | GGTTCAACTTCTGGCTCTGGGAAACCAGGAAGCGGCGAAGGGTCCACCAAGGGAAGCAC |
|  | 34-mer R | TTTAGTGCTACCTTCACCAGAACCAGGCTTACCTGAACCACTGGTGCTTCCCTTGGTGGACCC |
|  | YPet R3 | CCAGAGCCAGAAGTTGAACCAGCAGCAGTCAAAAATTCTA |
|  | ECFP F3 | CTGGTGAAGGTAGCACTAAAATGGTGAGCAAGGGCGAGGA |
| **Mutant** | S22A F | CACTCCGCTGGCGCCCACCCGCA |
|  | S22A R | CGGGTGGGCGCCAGCGGAGTGGA |
|  | S392A F | CCTGTCCCCCGCCCCTACCTCGCAG |
|  | S392A R | CGAGGTAGGGGCGGGGGACAGGCGT |
|  | R605A F | TTCATTGGTGCATCCGAGGATTG |
|  | R605A R | TCCTCGGATGCACCAATGAAAAA |

**Movie S1** (related to Fig 3). Lamin A pSer 22 dynamics during cell mitosis in a representative HeLa cell expressing LAPS.
